# The Protracted Maturation of Associative Layer IIIC Pyramidal Neurons in the Human Prefrontal Cortex During Childhood: A Major Role in Cognitive Development and Selective Alteration in Autism

**DOI:** 10.3389/fpsyt.2019.00122

**Published:** 2019-03-14

**Authors:** Zdravko Petanjek, Dora Sedmak, Domagoj Džaja, Ana Hladnik, Mladen Roko Rašin, Nataša Jovanov-Milosevic

**Affiliations:** ^1^Department of Anatomy and Clinical Anatomy, School of Medicine, University of Zagreb, Zagreb, Croatia; ^2^Department of Neuroscience, Croatian Institute for Brain Research, School of Medicine, University of Zagreb, Zagreb, Croatia; ^3^Center of Excellence for Basic, Clinical and Translational Neuroscience, School of Medicine, University of Zagreb, Zagreb, Croatia; ^4^Department of Neuroscience and Cell Biology, Rutgers University, Robert Wood Johnson Medical School, Piscataway, NJ, United States; ^5^Department of Medical Biology, School of Medicine, University of Zagreb, Zagreb, Croatia

**Keywords:** cerebral cortex, theory of mind, cortico-cortical neurons, dendritic development, schizophrenia, excitatory transmission, glutamate

## Abstract

The human specific cognitive shift starts around the age of 2 years with the onset of self-awareness, and continues with extraordinary increase in cognitive capacities during early childhood. Diffuse changes in functional connectivity in children aged 2–6 years indicate an increase in the capacity of cortical network. Interestingly, structural network complexity does not increase during this time and, thus, it is likely to be induced by selective maturation of a specific neuronal subclass. Here, we provide an overview of a subclass of cortico-cortical neurons, the associative layer IIIC pyramids of the human prefrontal cortex. Their local axonal collaterals are in control of the prefrontal cortico-cortical output, while their long projections modulate inter-areal processing. In this way, layer IIIC pyramids are the major integrative element of cortical processing, and changes in their connectivity patterns will affect global cortical functioning. Layer IIIC neurons have a unique pattern of dendritic maturation. In contrast to other classes of principal neurons, they undergo an additional phase of extensive dendritic growth during early childhood, and show characteristic molecular changes. Taken together, circuits associated with layer IIIC neurons have the most protracted period of developmental plasticity. This unique feature is advanced but also provides a window of opportunity for pathological events to disrupt normal formation of cognitive circuits involving layer IIIC neurons. In this manuscript, we discuss how disrupted dendritic and axonal maturation of layer IIIC neurons may lead into global cortical disconnectivity, affecting development of complex communication and social abilities. We also propose a model that developmentally dictated incorporation of layer IIIC neurons into maturing cortico-cortical circuits between 2 to 6 years will reveal a previous (perinatal) lesion affecting other classes of principal neurons. This “disclosure” of pre-existing functionally silent lesions of other neuronal classes induced by development of layer IIIC associative neurons, or their direct alteration, could be found in different forms of autism spectrum disorders. Understanding the gene-environment interaction in shaping cognitive microcircuitries may be fundamental for developing rehabilitation and prevention strategies in autism spectrum and other cognitive disorders.

## Quantitative Expansion of the Cerebral Cortex and Microcircuitry Changes: Role in the Appearance of Complex Human-specific Cognition

Increase in brain size, particularly an increase in the number of neuronal columns of the cerebral cortex, is the prerequisite enabling humans to achieve tremendous mental capabilities such as self-awareness, consciousness, language, abstract thinking, cognitive flexibility, mathematical abilities, as well as representational memory and complex social cognition (1–6). These abilities are not only species-specific features; the cognitive state achieved by humans represents a new qualitative level in mental functioning (7–9). It is correct that some animal species, in particular apes, are able to achieve a rudimentary level of some of these mental abilities (10–12). However, complex neuropsychiatric disorders as autism, schizophrenia or psychopathy are not present in any other species (13–17), which sets humans apart regarding cognitive and emotional features and capacities.

One of the most important human-specific abilities is complex social cognition, which includes processing, storing, and applying information about other people and social situations (18). Social cognition is the base for complex personal competencies, which are altered in the above mentioned diseases. From a neurobiological point of view, it is interesting that fundamental cognitive shift, which sets up human-specific cognitive abilities, the ability to understand the mental state of oneself or others (mentalization, i.e., “theory of mind”), appears in the period of transition from infancy to childhood (19–21). Humans and great apes (as our closest relatives) share roughly the same course of psychological development during the first 18 months of life (22). Around this age in both species, the brain nearly achieves adult neuronal composition, and even overall size (23–25). Nevertheless, in humans, intensive and diffuse changes in functional connectivity continue throughout the rest of childhood (26–31), while apes do not exhibit further important progress in cognitive capacities after the second postnatal year (22).

How did this unmatched shift in mental functioning between apes and humans appear without a robust quantitative increase in overall brain structure, i.e., overall increase in complexity of dendrites, or formation of new pathways and connections on most of the neurons? It should be noted, that a tremendous increase in the number of cortical neurons and connections, is a biological prerequisite to enabling high cognitive functioning (32–37). But at a certain point further quantitative expansion is not enough to initiate a more complex functional outcome, since the present pattern of organization does not allow proper integration inside numerically expanded circuitries. To make such an expanding system function properly, new microcircuitries that provide novel integrative properties are needed (31, 38–42). As such, the enhanced integration across cortical areas, along with an increase in network processing capacities, could come as a result of structural changes inside few selective microcircuitries (43–48).

**Figure 1:**
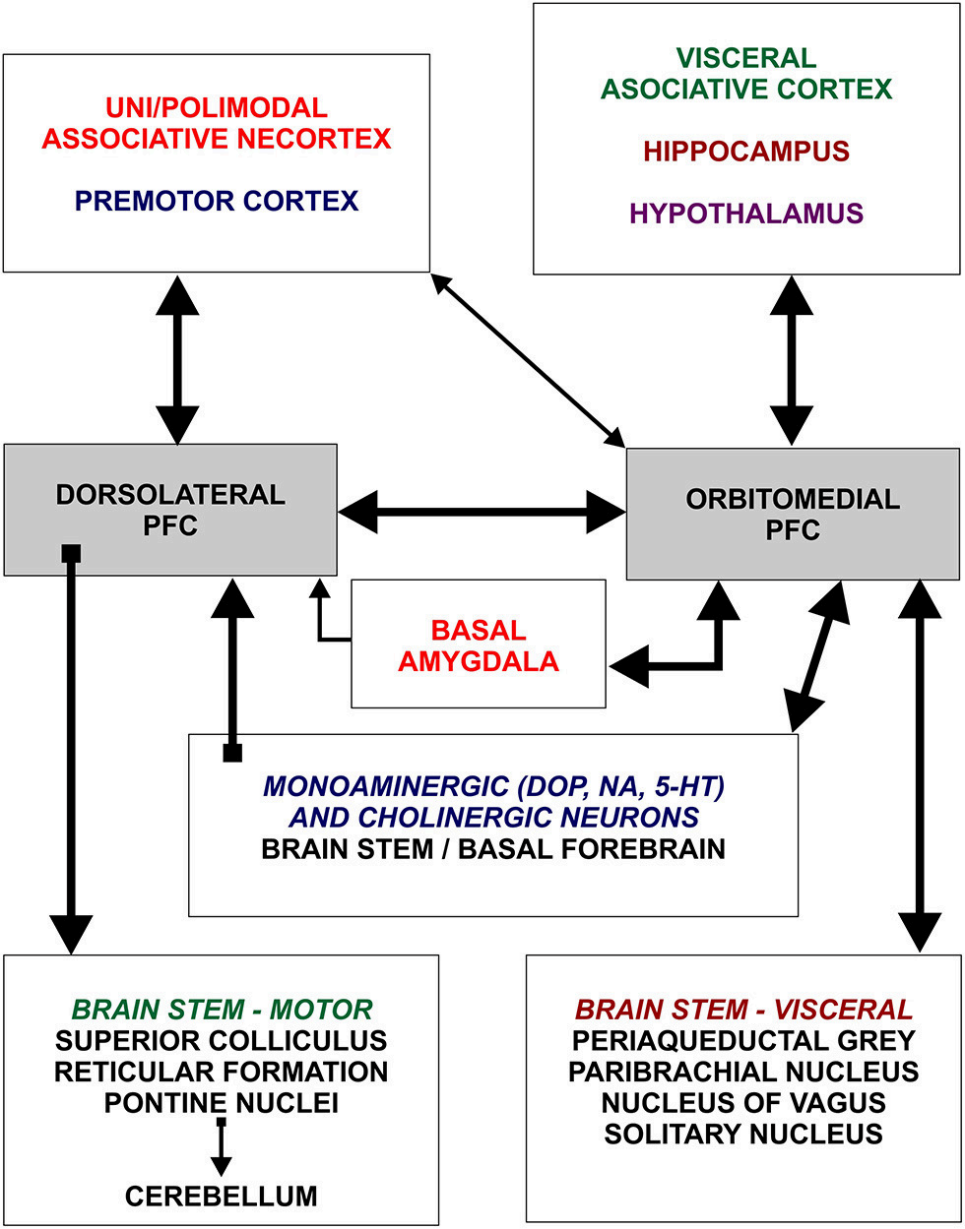
Schematic representation of two main subdivisions of the prefrontal cortex and general organization of their afferent and efferent connections. Dorsolateral prefrontal cortex establishes rich connections with all neocortical areas, except primary regions, whereas orbitomedial prefrontal cortex is mainly connected with hippocampus and cortical regions processing visceral information. Note that dorsolateral and orbitomedial prefrontal cortex are densely interconnected. Figure is based on Groenewegen and Uylings (49). DOP, dopamine; NA, noradrenaline; 5HT, serotonin.

It is possible that such changes are focused onto specific cortical areas. For example, the prefrontal cortex has abundant connections to most of the remaining cortical areas (49–53). Therefore, changes in functional properties of prefrontal cortex output (Figure 1) change the information processing throughout the whole brain (54). To produce considerable functional changes to the output, structural changes within the prefrontal cortex do not need to be “robust,” i.e., they do not need to include dendritic growth of a wide range of neurons. Even fine changes, e.g., growth focused on selective neuronal populations that have rich local connectivity, may cause dramatic changes in functional properties of the prefrontal cortex. Based on previous work by our and other research groups, we suggest that deep located large layer III(C) pyramidal neurons (L3N) of the human prefrontal cortex could perform such a role.

The L3N have a unique developmental pattern during early childhood (55, 56) that correlates with the appearance and boost in the maturation of cognitive abilities, such as self-awareness and complex social cognition. With a detailed overview of mature connectivity patterns, here we present a possible integrative role of L3N in cortico-cortical network processing. Their selective alteration in pathological conditions could produce immense changes in mental capacities, due to the failure of proper integration (57, 58). Therefore, we suggest that alterations in mentalization and communication abilities found in the autism spectrum and social communication disorder are a result of disrupted development of circuitries established by L3N (59).

## Molecular Features and Cytoarchitecture of Human Prefrontal Cortex: the Role of Associative Neurons in Inter-areal and Inter-columnar Connectivity

**Figure 2:**
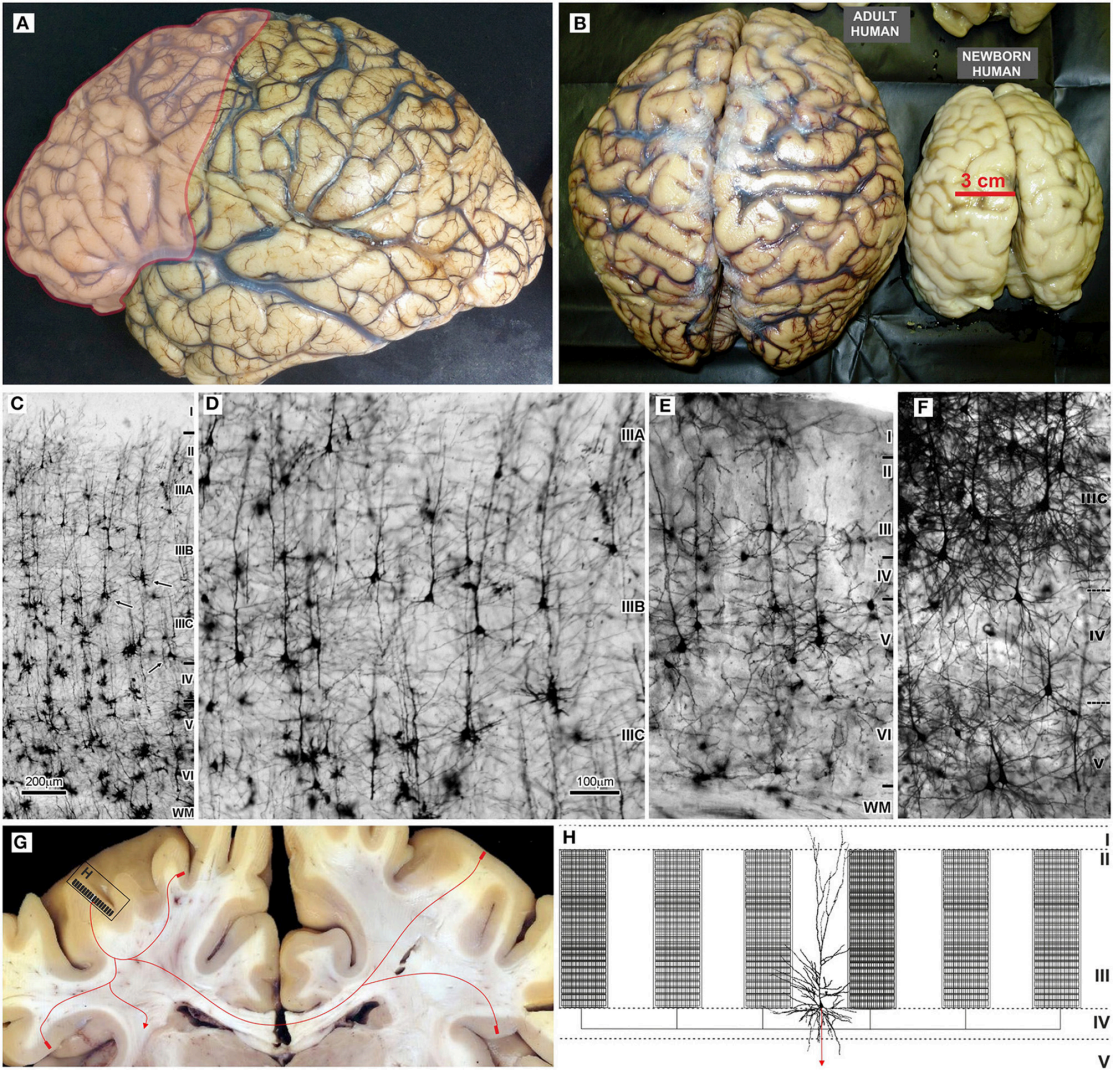
Macroscopic and microscopic features of the adult human brain, including schematic organization of extrinsic and intrinsic cortical projections of associative layer IIIC neurons. **(A)** In the adult human brain frontal granular cortex occupies 80% of the frontal lobe, and almost one third (red) of the total cortical surface. **(B)** Around the age of two, the brain is very close to its adult size (23). Microphotography of Golgi Cox **(C–E)** and rapid Golgi **(F)** impregnated sections of the associative areas in the human **(C,D,F)**, and mice neocortex **(E)**. **(G)** Large layer IIIC neurons are considered to be associative neurons, connecting several higher order areas in the ipsi- and contralateral hemisphere, with the columnar pattern of axon ramification (60, 61). Ipsilateral collaterals are much more numerous **(H)**, and around 80% of synapses are established within the area of origin. Local axon branches are forming numerous terminal ramifications which have columnar distribution through layers II and III, and extend several millimeters around the cell body (62–64). The figure is a compilation of figures published by Hladnik et al. (65) **(A,B)** and Džaja et al. (66) **(C–E)**. Scale bar 200 μm **(C)** and 100 μm **(D–F)**.

Evolution of the human brain (Figures 2A,B) is characterized by a sharp linear increase in the number of cortical neurons (67–69), but also with an exponential increase in the number of cortico-cortical projecting neurons (50, 67, 68). This leads to increased thickness of upper cortical layers (Figures 2C–E) which contain more neurons than lower layers populated by subcortically projecting neurons (3, 4, 39, 65, 66, 70, 71). So, the primate neocortex is characterized by a tremendous increase in the number of columns and changes to their internal neuronal composition (Figure 2F). Last but not least is the increase in width inside and between columns. The increase in width of columns is a result of increase in dendritic complexity of principal cells (72), i.e., more “space” between columns is a result of increased interconnectivity (73).

These changes are present in a majority of cortical areas, but are most prominent in several prefrontal cortex areas and related with their important integrative function inside cortico-cortical network (52, 53, 74, 75). The connectivity pattern of human prefrontal cortex (Figure 1), particularly its highly expanded dorso-lateral part, is characterized by massive reciprocal projections to both, multimodal and unimodal-parasensory associative areas (44, 76). This allows the prefrontal cortex to have a major role in regulating synchronous and coordinated activity between cortical areas. Experimental studies in monkeys and functional studies in humans have confirmed that the prefrontal cortex is functionally the highest associative region of the primate brain (38, 49, 51).

In humans, some areas of the prefrontal cortex show a specific cytoarchitecture, the magnopyramidality of layer III (77), i.e., deep located large layer III neurons exceed the size of large layer V pyramids. When compared to other populations of cortico-cortical projecting neurons, the L3N show strong acetylcholinesterase (AChE) (78–80) and SMI32 (antibody against phosphorylated protein H) reactivity (81–83) as a result of higher metabolic rate and prominent axonal tree. The density of SMI32/AChE reactive L3N, as well as their size and intensity of staining, is higher in human associative areas than in monkeys. In lower mammals, SMI32/AChE reactivity in deep layer III was not found (84).

The L3N are a subset of cortico-cortical neurons with long ipsi- and contra-lateral projections (Figure 2G) (40, 60, 61, 85). Individual L3N establish projections to several different areas suggesting a major role in inter-areal integration that grants them the title “associative” neurons (86). They are characterized by an astonishing number of intracortical axonal collaterals (Figure 2H), that extend around the cell, having dense columnar distribution of their terminal ramification through layers II and III (62–64). Thus, L3N are playing the major role in intercolumnar connectivity within a particular cortical area. In monkeys, L3N are indeed the key element for processing working memory and other prefrontal cortex-dependent high cognitive functions (87–90).

Above mentioned features of connectivity, functional properties and evolutionary expansion support the idea that L3N underlie the highly efficient network integration throughout the human cerebral cortex. We propose that selective structural and molecular changes of associative L3N in the human prefrontal cortex around the age of 2 and several upcoming years (55, 56), change the properties of the whole prefrontal cortex output, and have a pivotal role in cognitive maturation characterizing the preschool period. Developmental changes selectively related to this neuron class may be crucial for the appearance of cognitive abilities needed for the understanding of higher levels of inter-personal interaction, and to lay foundation for a further increase in cognitive capacity observed later throughout the childhood and adolescence, that ultimately leads to socio-emotional maturity. Selective alterations of the L3N were described in neuropathological states characterized by intense and global changes in the efficiency of the cortical network (91–96). We propose that selective alterations of associative L3N have a pivotal role in the “dis-connectivity” of prefrontal cortex found in autism spectrum disorder (97, 98), but also in other prefrontal cortex-associated disorders, like schizophrenia (90, 99, 100). These two disorders share similarities in cognitive pathology, and are characterized by global cortical dysfunction, without concurrent structural alterations and specific structural pathology identified so far (16, 17).

## Understanding the Mental States of Oneself or Others: Neuronal Development of Prefrontal Cortex During Early Childhood and Focal Disconnectivity in Autism

The capacity to attribute mental states (mentalization) remains one of the quintessential abilities that makes us human and is defined as the “theory-of-mind” (ToM) (20, 21, 101, 102). This ability appears during the second year of life and subsequently sophisticates through childhood with cognitive spurts at specific time points (103). Following temporal pattern at which various levels of ToM have been achieved is important since it reflects changes inside cortical circuitries which allow shifts in mental capacities.

Most data marks the infant to child transition as a point when first ToM abilities appear, i.e., during the second year of life. Infants around 12–15 months of age display behaviors that are prerequisites to ToM development (104–106). Nevertheless, it is difficult to talk about internalization of mental abilities before 18 months of age (107, 108). By the age of 2, children are clearly aware that there is a difference between thoughts in their mind, and things in their surroundings (109). An average 3-year-old knows that the brain has a set of mental functions, such as dreaming, wanting, thinking, and that different persons may want, like and feel different things. Further important cognitive twists occur around the age of 4, when children realize that thoughts might not be true (110). Also, a 4-year-old can remember that their own belief has changed which is not the case with a 3-year-old (111). By the end of early childhood (ages 5 to 6), children realize that people talk and act on the basis how they think the world is, even when it does not reflect the reality of the situation. They can keep secrets and understand that sometimes a person may believe something that is not true, and that what a person does or says, can be based on a false belief (112, 113).

The cortical areas related to ToM tasks typically activate the frontal lobe. In particular, neuroimaging studies of ToM showed activations in the dorsal prefrontal cortex (Brodmann area 9). However, other frontal regions were also involved in understanding and controlling oneself, as well as in interaction of thoughts about oneself and others (112, 114–117). Therefore, the prefrontal cortex can be considered as a region with a key role in social cognition, and it is assumed that pathological substrates in states characterized by disrupted social cognition, such as psychopathy personality (14) and autism spectrum disorder (59, 118), must be located within.

In autism spectrum disorder (ASD) and related social (pragmatic) communication disorders, social interactions are affected (119) due to difficulties in the aptitude for inferring other people's states of mind, such as intentions, beliefs, desires and wishes (120). When a false-belief test is applied to children with ASD, most of them fail even at the age of 11, in contrast to typically developed children who pass the test by the age of 4 (121). In a modified (122) and simplified (123) form of the false belief task, typically developing children show ToM abilities latest by the age of 2.5, while many of them show it already at the age of 1.5 years (107, 124). These abilities are lacking in ASD subjects, showing that deficiency in the ability to reflect on the contents of one's own and other's minds (101, 125) is a core cognitive feature of ASD. This lack is a consequence of a disturbed cognitive development at some point during the period of early childhood (1.5–6 years) (126–128). In search for neurobiological correlations, it is important to recognize that cognitive impairment in ASD is specific and different from learning difficulties of blind or deaf people (129–131).

Neuroimaging data show that dorsolateral and medial prefrontal cortex in ASD are hyperconnected during the second year of life (132–137). In parallel with hyperconnectivity, many of ASD individuals undergo brain overgrowth that is particularly pronounced in the frontal lobes (138–141). The hyperconnectivity later on changes to hypoconnectivity (142–144). Therefore, it is still debated if ASD should be considered as a disorder characterized by hyper- or hypoconnectivity (145, 146).

Nevertheless, the concept of hypo- or hyperconnectivity seems to be oversimplified. Neuroimaging data are in line with the view that ASD symptomatology is the result of disconnection in areas involved in the processing of language, executive and socioemotional reaction as well as in abstract and conceptual thinking. Furthermore, there is a disconnection in cortical regions that are highly evolved in humans and involve higher-order associative processing along with the prefrontal cortex (147–151). Alteration in ASD was also found in many regions of the temporal lobe and in adjoining parts of the occipital and parietal lobes, including the insula and regions important for ToM processing (117, 125, 152, 153). However, the majority of functional and structural connectivity studies in ASD suggest that the key disconnection must be between the frontal lobe and other higher order association cortices (154–160), and that the frontotemporal, frontolimbic, frontoparietal, and interhemispheric connections are altered. In addition, alterations in synaptic organization related to specific deficits were found (161–166). The type and range of cognitive pathology in ASD suggests that structural alterations are focused, as well as delicate, but present even among highly-functional adults with ASD (122, 167–169). Preservation (or even enhancement) of other mental functions (170, 171) suggests that development of certain circuitries is spared, supporting the model of “focal disconnections” which appear during development. Therefore, ASD should be considered as a form of “developmental disconnection syndrome” (172–174).

In conclusion, structural and functional data in ASD suggest that development of specific micro-circuitries is disrupted during the ToM acquiring stage of infant to child transition (second year of life), or in milder ASD forms during the upcoming years (118, 120, 175, 176). The role of distinct neuron classes in the prefrontal cortex for processing ToM and complex social cognition is not yet defined, and therefore the neuronal correlates of ASD pathology remain unknown (177).

Based on connectivity patterns, as well as the pattern of their development and maturation, selective changes of associative L3N microcircuits in the prefrontal cortex could represent one of the major biological substrates for normal cognitive development during early childhood. Consequently, abnormal L3N development could be associated with appearance of ASD symptomatology.

## Sequential Differentiation of Principal Neurons in the Prefrontal Cortex And Early Development of Cortico-cortical Microcircuitry

### Mechanism of Dendritic Growth

**Figure 3:**
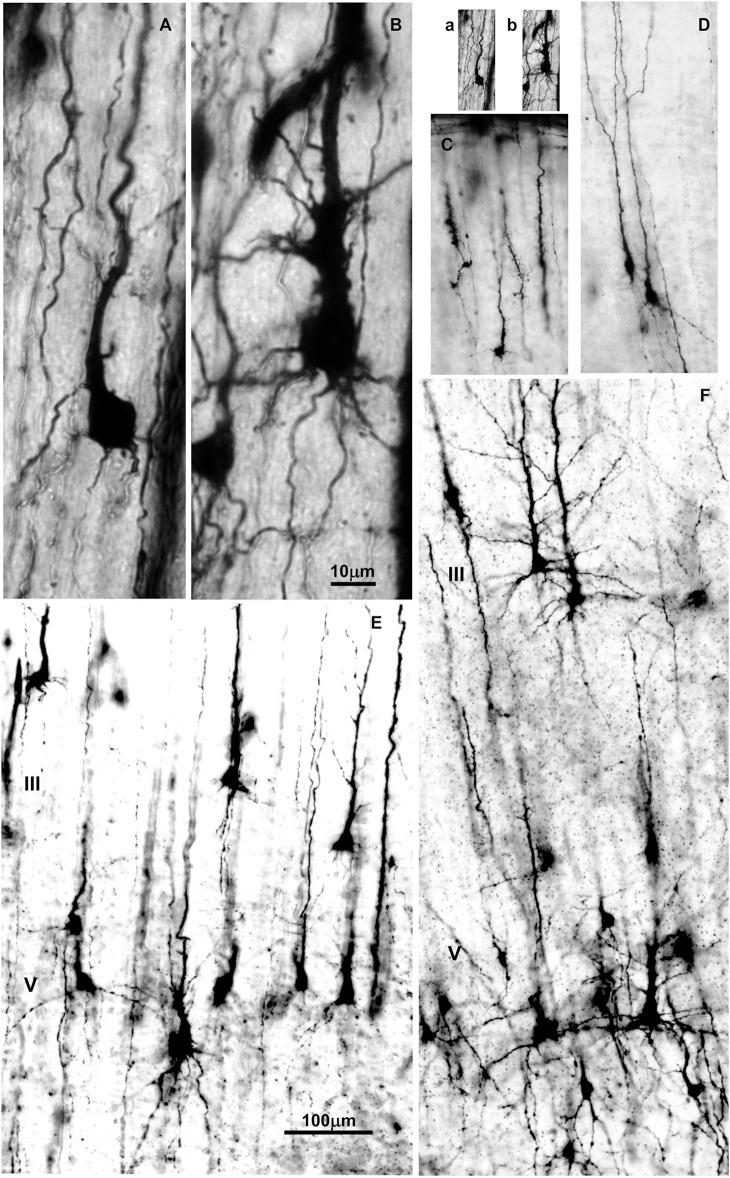
Changes in dendritic morphology of rapid-Golgi impregnated pyramidal neurons in the dorsolateral part of the prefrontal cortex during the second half of gestation **(A–D)** and first postnatal month **(E,F)**. Microphotography of rapid Golgi impregnated sections in the human fetal prospective dorsolateral prefrontal cortex at 21 **(A,B)**, and 32 postconceptional weeks **(C,D)**, newborn **(E)** and one month old infant **(F)**. Scale bar 10 μm **(A,B)** and 100 μm **(C–F)**. **(a,b)** are **(A,B)** shown at the same magnification as **(C–F)**.

Development of dendrites is one of the essential processes in differentiation and maturation of neuronal circuitry (178–182). Developmental changes in dendritic size and complexity will define the total neuronal receptive field. Since dendritic development occurs in parallel with rapid synaptogenesis (183) and axon growth (184, 185), it will affect both the neuronal functional response to the input and the neuronal output (186). Dendritic development typically undergoes three phases:

The first phase of dendritic growth starts after the neuron arrives to its final position within the cortical plate (37, 187, 188). This phase is characterized by the protrusion of primary basal dendrites and apical dendrite, which arise from the cell body, including appearance of oblique dendrites which grow out on the proximal site of the apical dendrite (189). No significant outgrowth of additional branches on primary oblique and basal dendrites occurred during this phase (Figure 3).

**Figure 4:**
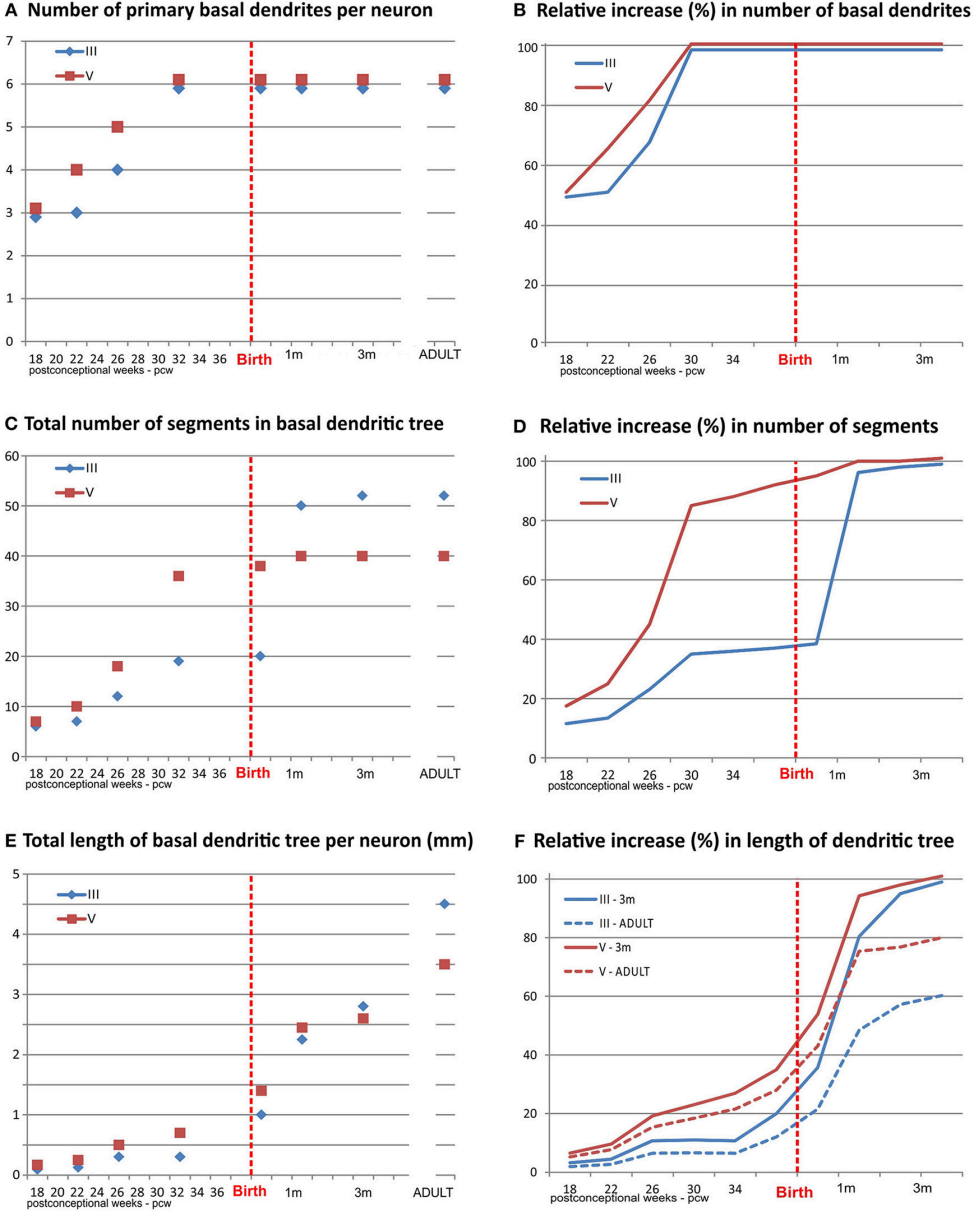
Graphical presentation of quantitative data from the basal dendritic tree of large layer III and V pyramidal neurons in the human dorsolateral prefrontal cortex, impregnated by a rapid Golgi method, in a period of rapid growth between 18 postconceptional weeks, and third postnatal month, compared to adult values. An outgrowth of primary basal dendrites **(A)** started earlier on layer V pyramidal neurons, during the middle trimester of gestation prior to layer III, but not later on **(B)**. The number of basal dendritic segments (indicating frequency of bifurcation) shows a clear inside-out gradient until birth **(C)**. A constant, slow outgrowth of new segments is present, for both classes, during the middle trimester of gestation, followed by rapid increase in period 26–32 PCW for layer V pyramidal neurons **(D)**. The major outgrowth of new segments occurred for layer III pyramids during the first postnatal month. No additional segment outgrowth is observed after first postnatal month. Despite rapid segment outgrowth up to the 32 postconceptional weeks, the increase in total length for layer V pyramids **(E)** was rather slow. Most of the elongation occurred later, during the last 2 months of gestation and first postnatal month **(F)**. At the same time, opposite to layer V, a considerable increase in length occurred for layer III during the period of rapid segment outgrowth. At the 3rd postnatal month, layer III rapid Golgi impregnated neurons have just exceeded 50%, whereas layer V pyramidal neurons exceed 80% of their adult length (dashed lines). Data shown here were extrapolated from the studies of Mrzljak et al. (190) and Petanjek et al. (55).

The second phase is characterized by extensive and rapid growth of the dendritic tree. Initially, new segments grow out on the, during first phase formed primary basal and oblique dendrites (Figures 4A,B). This is followed by an increase in the size of the dendritic tree achieved mainly through elongation of present branches. Importantly, the appearance of functional glutamate receptors is crucial for inducement of rapid dendritic growth (183, 191–195). This strongly supports the view that ingrowth of glutamatergic thalamo-cortical and cortico-cortical afferents during the fetal and perinatal period, triggers the rapid dendritic growth of principal neurons (196).

The final stage of dendritic growth (once up to 20% of total dendritic length is established) is characterized by significant, but much slower elongation of dendrites than during the second phase. Many connections established on developing dendrites are functional at the beginning of this stage, making dendritic development more sensitive to environmental influences (“nurture”). In contrast to the second phase, glutamatergic activity during the third phase stabilizes the dendritic tree instead of promoting its growth (199–201). As such, the last stage of dendritic development is the longest and is characterized by a slighter increase in the length of the dendritic tree (202, 203).

### Intensive Perinatal Dendritic Growth of Associative Neurons Results in Early Functional Microcircuitries

Dendritic development and synaptic rearrangement of principal neurons have been extensively studied in the monkey and human prefrontal cortex (189, 190, 197, 199, 204–221). In the human fetal prefrontal cortex, intensive dendritic growth (second phase) of both deep layer III and V principal neurons, starts 12–15 weeks after they arrived into the cortical plate. However, beginning of the second phase differs between these two subpopulations of principal neurons. For the large layer V pyramidal neurons it coincides with the ingrowth of thalamo-cortical fibers into the cortical plate by the end of the middle trimester of gestation (188, 196, 222, 223). In contrast, intensive growth of the L3N begins with ingrowth of cortico-cortical fibers by the end of the last trimester of gestation (224, 225). Thus, for the two main classes of large pyramidal neurons in the human prefrontal cortex there is an inside-out gradient of differentiation during the prenatal period, and intensive growth seems to be induced by the arrival of specific glutamatergic afferents.

At birth, large layer V principal neurons have already attained their adult dendritic complexity (branching pattern), while L3N are not well developed (190), suggesting that the cortico-cortical network is not highly functional at that time (226–228). Indeed, the most intensive dendritic development of the L3N is the first postnatal month, when around 60% of basal dendritic segments appear (Figures 4C,D), and almost half of the total size is achieved (Figures 4E,F). Thus, the L3N dendritic tree “catch-up” large layer V pyramidal neurons in absolute values of their length and complexity soon after birth. By the third postnatal month both classes are equal in total dendritic tree length, and have achieved not only adult complexity, but also, adult-like overall dendritic three shape.

The maturity level of layer V principal cells reached soon after birth is not surprising, as these cells are key neuronal elements for processing early executive functions of the prefrontal cortex (43, 229–231). However, the maturity level of L3N reached between first and third postnatal month is somewhat surprising (55, 56), as these cells are believed to be the key elements involved in sophisticated, evolutionarily recent, and human-specific cognitive functions that develop later on (232, 233). Such an early functioning cortico-cortical neuronal network centered on L3N may represent a neurobiological basis for cognitive functions present already in the first months after birth (234–238). Behavioral and functional studies found that the perinatal period (32 week of gestation−3 months postnatal) is characterized by rapid transformation and disappearance of fetal patterns of behavior, but also with concomitant appearance of certain aspects of cognitive functions, which will intensively develop through infancy (124, 239–246).

### Sequential Development of Microcircuitries in the Human Prefrontal Cortex During the First Postnatal Year

**Figure 5:**
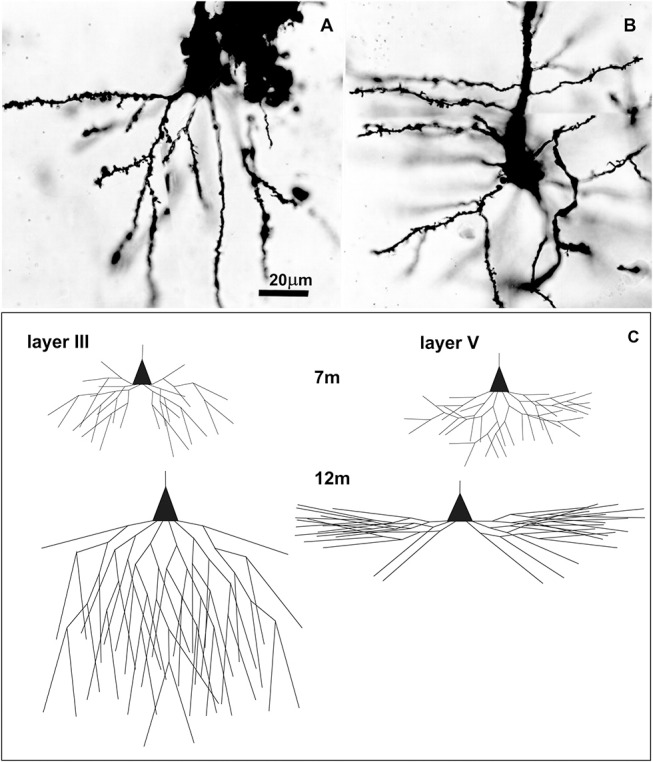
Golgi Cox impregnated pyramidal neurons in human Brodmann area 9 at 7 postnatal months and changes in basal dendritic tree between 7 and 12 months. Microphotography of Golgi Cox impregnated large pyramidal neurons in deep layer III **(A)** and layer V **(B)** at 7 postnatal months at higher magnification. Schematic drawings **(C)** indicating changes in length and complexity of dendritic tree of Golgi-Cox impregnated neurons between 7 and 12 months, showing that major dendritic growth for those two populations of neurons, occurred during the second half of the first postnatal year. A total number of segments is approximated on the basis of neuronal reconstructions (197), and percentage of segments cut at a particular dendritic order (198).

Not all classes of principal neurons in the prefrontal cortex undergo intensive dendritic growth during the prenatal and perinatal period as observed for large pyramidal neurons impregnated with the rapid Golgi method. Subpopulations of pyramidal neurons impregnated by the Golgi Cox method (197, 207) undergo a major dendritic growth after birth, mainly during the second half of the first postnatal year (Figure 5). Different modification of Golgi methods have a selective affinity to stain different neuronal subpopulations, i.e., the rapid Golgi method is more prone to impregnate large pyramidal cells. These differences in timing of intensive dendritic growth between different subclasses of principal neurons (247, 248) suggest that there is a different gradient of maturation for different subclasses of neurons, even within the same layer.

**Figure 6:**
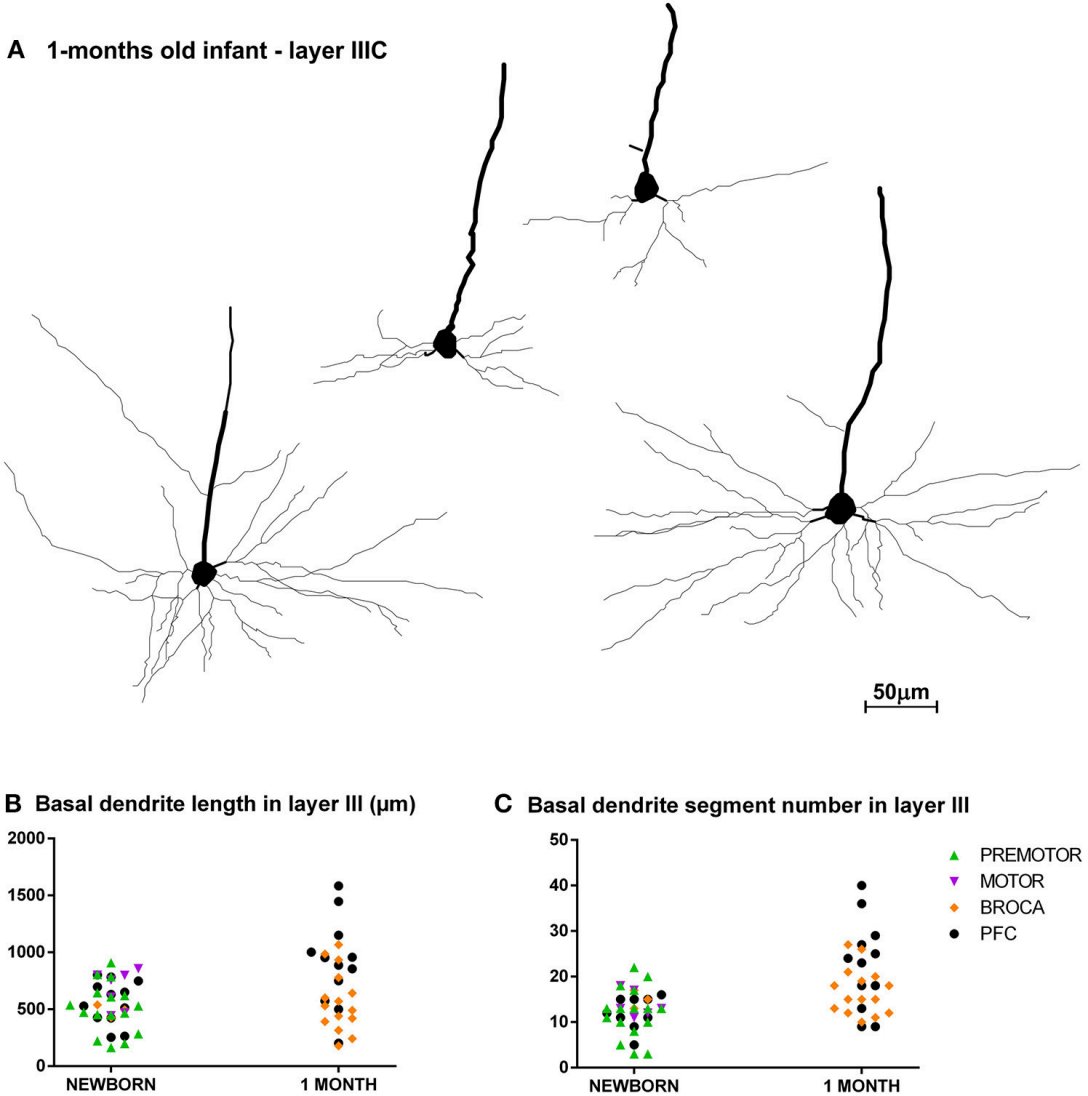
Asynchronous maturation of pyramidal neurons in layer IIIC during first postnatal month. **(A)** Rapid Golgi impregnated deep layer III pyramidal neurons in Brodmann area 9 of a 1-month-old infant in the prefrontal cortex show different stages of differentiation. Quantitative analysis of newborn and 1-month-old infant reveals large differences in total length **(B)**, and number of segments **(C)** in basal dendritic tree of deep layer III pyramidal neurons, across different areas of the human frontal cortex. Each symbol represents mean values per individual neurons.

In our recent work, by using rapid Golgi method and encompassing broader population of the layer III impregnated principal neurons (Figure 6), we showed significant differences in the level of dendritic differentiation during the first postnatal month within frontal lobe, that includes dorsolateral part and Broca's region as well as primary motor and premotor cortices (249). The populations of L3N attained a highly developed dendritic tree in all analyzed areas, whereas dendrites of other principal neurons in layer III were less differentiated. Such findings show an asynchronous maturation of different microcircuitries throughout the cortico-cortical network: some of them reach functional level soon after birth, while others are still very immature (47, 250–252). This is in contrast to the traditional view of hierarchical neuronal development across the cerebral cortex, which suggests a sequential gradient of maturation from lower to higher order areas. We propose that there is a sequential maturation of distinct elements, forming cortico-cortical circuitries across all frontal areas (88). Such a pattern of development may represent a neurobiological basis for the sequential development of cognitive functions during the first and second postnatal year (124, 235, 253–256). Also, rapid dendritic growth is related with maturity of glutamatergic and GABA-ergic receptors (257, 258), making dendritic differentiation more prone to environmental influences. As such, for the development of early maturing neurons, as is the case for L3N, already during the perinatal and early postnatal period, the environment has an important role in regulating their morphological differentiation (259–263).

## Selective Maturation of the Associative Large Layer IIIC Neurons During Early Childhood And Protracted Development of “Cognitive” Microcircuitry Through Adolescence

### Unique Pattern of Dendritic Growth of Associative Neurons in Human Prefrontal Cortex

For most subpopulations of principal neurons in the prefrontal (197, 207, 208, 218, 219, 221, 264, 265), and other regions of the human cerebral cortex (266–276) major postnatal dendritic growth occurs during the first year in parallel with massive synaptogenesis (185, 205, 206, 226, 277–284).

**Figure 7:**
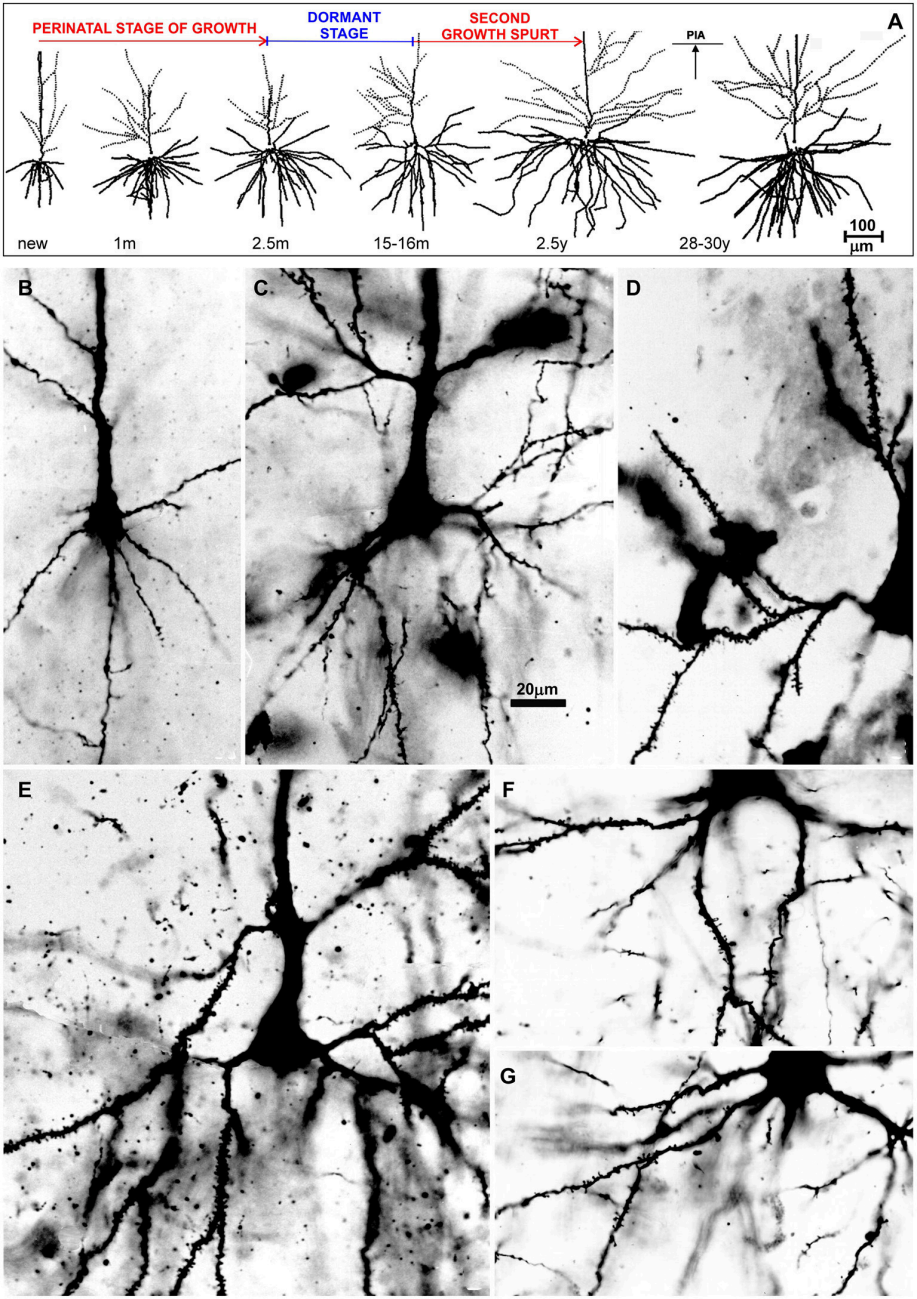
Postnatal development of rapid Golgi impregnated large layer IIIC pyramidal neurons in the magnopyramidal area 9 of the human prefrontal cortex. Three-dimensional reconstructions of basal and apical dendritic trees of rapid Golgi impregnated pyramidal cells in layer IIIC, projected onto the coronal plane **(A)**. Orientation toward the pia is indicated by the arrow. Oblique dendrites originate from the apical dendrite, and are represented by dashed lines. All layer IIIC pyramidal cells are represented at the same magnification (scale bar 100 μm) and at the following ages: newborn, 1-month-old, 2.5-months-old, 15-months-old infants, 2.5-year-old child, and 28-year-old adult. Dendritic trees of layer IIIC pyramidal cells increased between 16 months and 2.5 years of age. Note that there are no obvious differences between layer IIIC pyramidal cells of 2.5-month-old and 16-month-old infants (dormant stage), as well as 2.5-year-old and 28-year-old subjects. Microphotographs showing changes in morphology of rapid Golgi impregnated layer IIIC pyramidal cells of the Brodmann area 9 between: newborn **(B)**, infants aged 1 **(C)** and 16 **(D)** months, 2.5-year-old child **(E)**, 19-year-old **(F)**, and 73-year-old **(G)** adults (the magnification is same for all microphotographs; scale bar –20 μm). Even in these high-power microphotographs, the increase in dendritic complexity (an outgrowth of new segments) between newborn **(B)**, and 1-month-old infant **(C)** is obvious. The figure is taken from Petanjek et al. (55) with permission.

**Figure 8:**
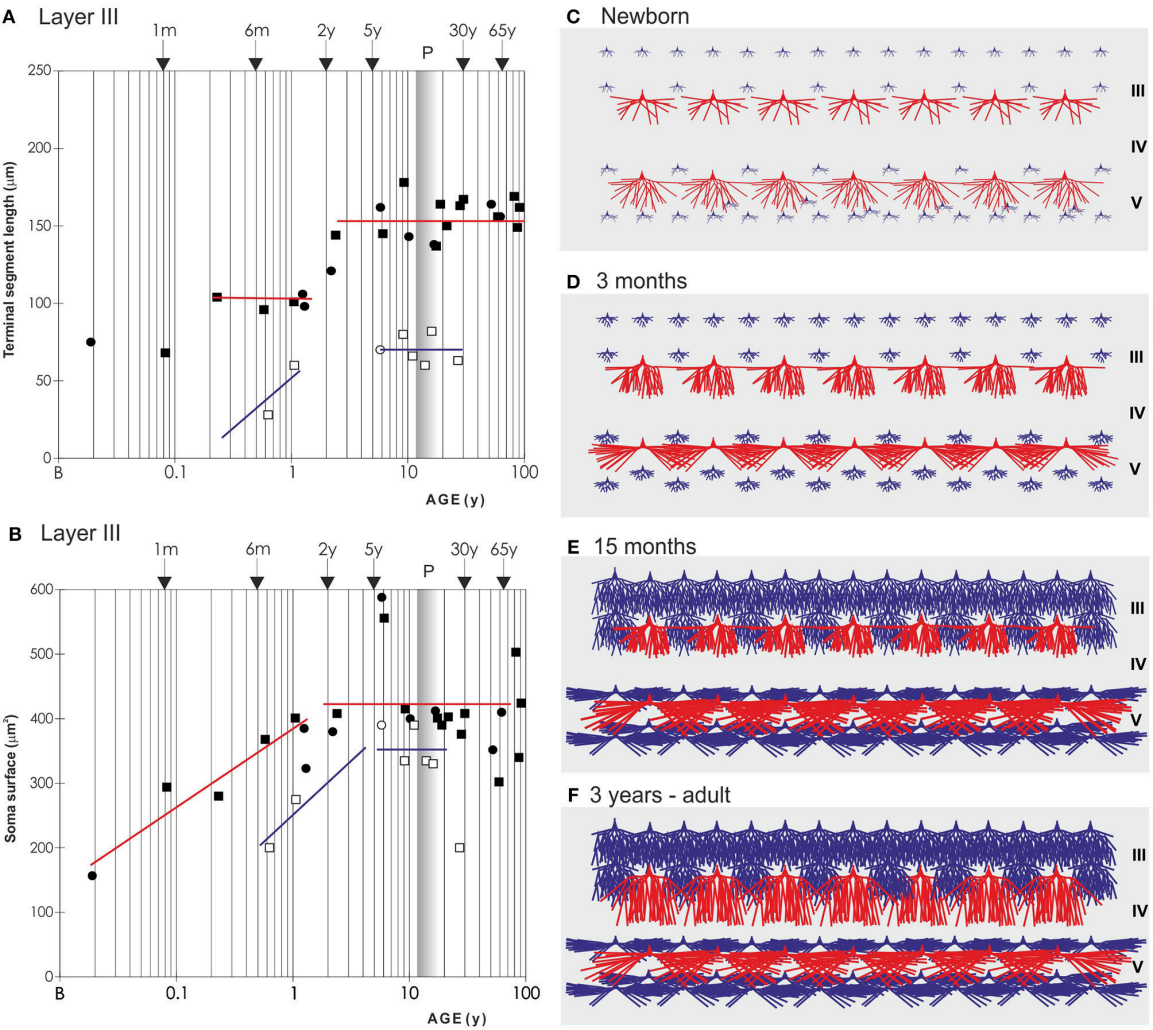
Graphical and schematic presentation of quantitative morphological data from basal dendritic tree of deep layer III and layer V pyramidal neurons, in magnopyramidal area 9 of the human dorsolateral prefrontal cortex impregnated by the Golgi method during postnatal development. Changes in terminal segment length **(A)** and soma size **(B)**, from birth until 91 years of age on layer III pyramidal neurons impregnated with rapid Golgi (full marks—red line), and Golgi Cox method (open marks—blue line). Data about terminal segment length **(A)** are shown here as they participate with 90% in the total length of dendrites. Data from rapid Golgi stained slides, regarding terminal segment length confirm, that the period between 3 and 16 months, is a dormant stage in basal dendritic growth for layer III pyramidal neurons, and that further growth occurs between 16 months and 2.5 years (at least 1/3 of total length). Large temporal overgrowth in cell body size was present in subjects 5 to 6 years old **(B)**. Data shown here were extrapolated from Koenderink et al. (197) and Petanjek et al. (55) studies. Squares represent males and circles represent females. The age is shown in postnatal years on a logarithmic scale. Puberty is marked by a shaded bar. Schematic drawings **(C–F)** of changes in length and complexity of dendritic tree of deep layer III and layer V pyramidal neurons, illustrate changes occurring in the cytoarchitecture and overall neuronal morphology. Total number of segments is estimated on neuronal reconstructions (based on real data) (55, 197) and values of missing dendrites were calculated by formula (198). Red represents rapid-Golgi impregnated neurons, and blue Golgi Cox impregnated pyramidal neurons. Illustrations for Golgi Cox neurons at newborn **(C)** and 3-months-old infant **(D)** stage are prediction based on dendrite growth pattern in the period 7–15 months. By the 15 months **(E)** most of the neurons have achieved an adult level of dendritic size, except large layer IIIC pyramidal cells impregnated by the rapid Golgi method **(F)**.

**Figure 9:**
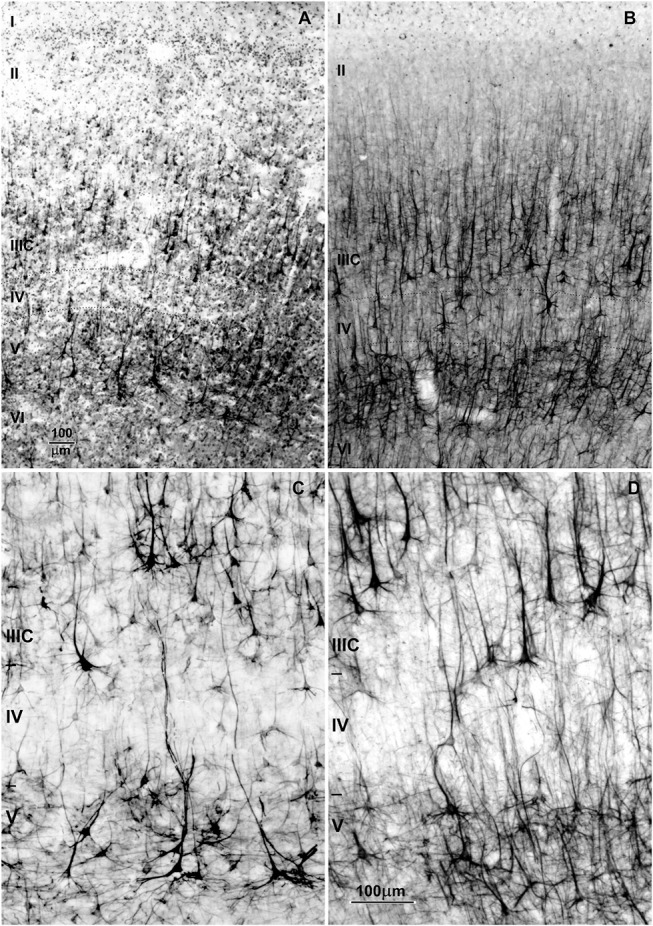
Neurofilament staining (SMI32) in Brodmann area 9 of child and adult human cortex. Microphotography of SMI32 (non-phosphorylated filament H) stained sections in Brodmann area 9 of the human prefrontal cortex at the age of 3 **(A,C)** and 38 years **(B,D)**. The deep part of layer III and layer V are sub-laminas most densely populated with reactive neurons. There are no differences in staining intensity of layer V neurons at both ages, however in the adult subject layer III, the amount of intensely stained pyramidal neurons with clearly visible dendrites has increased. Accumulation of neurofilaments in dendrites is corresponding to the length and complexity of axon, suggesting that pyramidal neurons located deeper in layer III continue with axon growth after the age of 3. Scale bar at 100 μm **(A–D)**.

An important exception from the typical temporal pattern of dendritic growth (see previous chapter) are associative L3N in the prefrontal cortex (Figures 7, 8). The L3N do not undergo the typical third stage of dendritic development like large layer V pyramidal neurons (55, 56). The layer V neurons attain more than 80% of their adult dendritic length by the third postnatal month. They then continue with further elongation during the third stage of dendritic development for roughly 1 year, and reach adult values around 15 months of age. In contrast, by the third postnatal month dendritic size of the L3N has reached only half of their adult values (Figures 7A–C, 8A,C,D). In addition, basal and oblique dendrites of the L3N have no significant growth (“dormant” period) until the middle of the second year (Figures 7A,C,D, 8A,D,E). Between 16 months and 2.5 years, length of L3N basal and oblique dendrites almost doubled, with growth rate higher than expected for the third stage (Figures 7A,D,E, 8A,E,F). To the best of our knowledge, this second L3N dendritic growth spurt represents an undescribed developmental feature for any class of cortical neurons. In the following period from 2 to 5 years, there is a further increase in synaptic spine density (285) at L3N dendrites, accompanied with molecular changes of this class of neurons. The L3N start to express strong AChE (78, 79) and SMI-32 (82, 265, 286) reactivity in their bodies and proximal apical and basal dendrites. This unique expression sets them apart from other classes of cortical neurons (Figure 9). Additionally, by the age of 5, the L3N show intensive Nissl staining paralleling transient somatic overgrowth (Figure 8B) (287–290).

Thus, the L3N acquire a significant portion of their maturity after infancy. We suggest that morphological and molecular changes on dendrites of the L3N in the period from 2 to 6 years are related to the growth and synaptogenesis of their own local intracortical projections, which establish very dense innervation on all cortico-cortical projecting neurons (62–64). Thus, changes in L3N intracortical projections will affect function of all prefrontal cortex neurons that project to other cortical areas. Consequently, L3N changes will be reflected on network processing throughout the whole cerebral cortex. Intracortical projections in experimental studies on rhesus monkey were found to be the last maturing part of the cortical excitatory network (209), whereas basic architecture for cortico-cortical projections is established earlier during infancy (41, 124, 243, 291–294). This leads us to conclude that large scale functional changes in the cerebral cortex, starting around the age of 2, are related with maturation of excitatory intracortical connections. Still, further maturation of cortico-cortical projections could not be excluded (116).

### Protracted and Environmentally Driven Synaptic Pruning of Associative Microcircuitries

**Figure 10:**
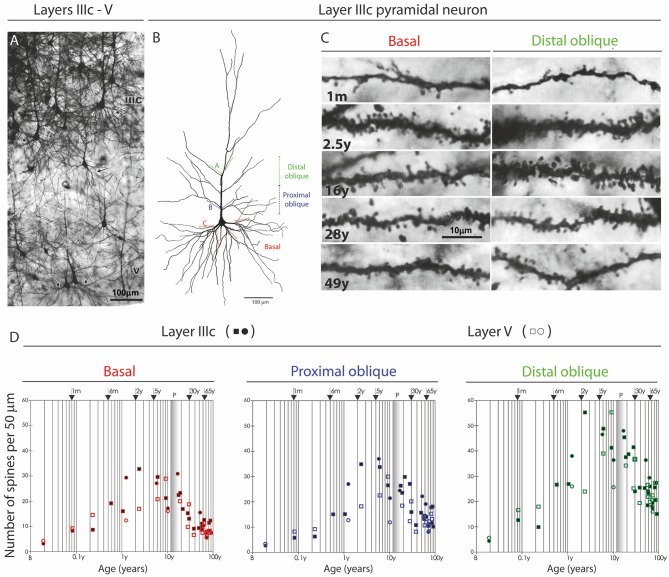
Changes in dendritic synaptic spine density on rapid Golgi impregnated large layer IIIC and layer V pyramidal neurons in magnopyramidal area 9 of the human prefrontal cortex from birth until 86 years of age. **(A)** Representative low magnification photographs of the rapid Golgi-impregnated layer IIIC and V pyramidal cells in the dorsolateral prefrontal cortex of a 16-year-old subject. **(B)** Neurolucida reconstruction of layer IIIC pyramidal neuron of a 49-year-old subject, illustrating dendrites selected for counting spines. **(C)** Representative high power magnification images of layer IIIC pyramidal neuron dendrites. **(D)** Graphs represent changes in spine numbers per 50 μm of dendritic length. The age is presented in postnatal years on a logarithmic scale. Puberty is marked by a shaded bar. Squares represent males, circles females. P, puberty; B, birth (fourth postnatal day); m, months; y, years. Figure published by Petanjek et al. (285).

Structural changes through the cortical network are not finished by the age of 5–6 years, while the circuitry reorganization continues throughout the rest of childhood and adolescence (28, 33, 36, 76, 90, 205, 216, 283, 295–317). Molecular tuning of synaptic strength during development, when synaptic numbers exceed adult values, is proposed to be a major mechanism for the environmental effect on circuitry reorganization. The period of overproduction and elimination of supernumerary synaptic spines corresponds to the developmental stage when principal neurons have the highest magnitude of plasticity (185, 201, 260, 277, 304, 318–326). In the prefrontal cortex, the stage of developmental plasticity is highly prolonged and extends even up to the third decade of life (Figure 10). Concomitantly, there is a prolonged peak in expression of genes regulating neuronal development, including those associated with schizophrenia (298, 307, 327, 328). The comparative analysis of mRNA expression in the prefrontal cortex shows that in the human brain, relative to non-human primates, the dramatic changes in transcriptome profiles are delayed (283, 304, 307, 329, 330). So, extraordinary protracted circuitry reorganization is a specific feature of human higher-order associative areas.

It is possible however, that distinct types of microcircuitries may have different rates of synaptic formation and elimination. The pruning of supernumerary dendritic spines during the third decade of life is most pronounced and protracted on the L3N. It starts earlier on segments that are targeted by thalamo-cortical, rather than on those targeted by cortico-cortical projections (285). Studies obtained in monkeys and humans show somewhat higher synaptic overproduction in supragranular (including L3N and cortico-cortical projecting neurons) vs. infragranular (including layer V and subcortical projecting neurons) layers (205, 226). Regional differences in the number of grown and pruned spines on the layer III neurons have been described in the monkey and human cortex, with highest spine overproduction in the prefrontal cortex and lowest in the primary sensory regions (208, 221, 250, 279, 282, 331). The level and duration of synaptic overproduction, and consequently the level and duration of developmental plasticity (332), increases within increasing functional hierarchy of distinct microcircuits. Thus, microcircuits that are processing the highest cognitive functions, such as social abilities, are subject to the highest developmental remodeling induced by psycho-social and emotional environment (32, 39, 250, 252, 260, 305, 333, 334).

## The Development of Prefrontal Cortex Associative Neurons in Autism: a Model of Selective Neuronal Vulnerability in Global Cortical Network Disconnectivity

Based on the above, we suggest that selective disruption of L3N could cause global cortical network disconnectivity, underlying ASD cognitive symptomatology. The protracted and biphasic pattern of L3N dendritic growth, coupled to the intensive molecular maturation after infancy, is not described for any other population of principal neurons. This developmental timing overlaps with the period when specific ASD symptomatology becomes evident.

Thus, alteration of the specific neuronal population with “strategic” position inside cortical circuitry, like L3N, could lead to global cortical network dysfunction. The timing of appearance and severity of symptoms in ASD might depend on the affected level of structural and molecular maturation of associative neurons during early childhood (2–6 years). For example, if development of the L3N is affected during the second year of life, it is most likely that the elongation of dendrites would be altered. This possible reduction in dendritic size would result in abnormal input on the L3N, and would change their functional properties. Consequently, as the L3N densely innervate all layer II/III cortico-cortical neurons, a robust alteration in dendritic morphology and consequently possible out-growth of intracortical projections of L3N, would lead to disrupted inter-columnar processing (335). We would expect such changes in subjects with a more serious form of ASD, where cognitive pathology already emerged during the second year of life.

In some ASD cases, specific cognitive symptoms are not apparent during the second, and even third year of life. Those subjects only show a subtle deflection of ToM, with no signs of any other neurological or psychiatric comorbidity, like in Asperger's syndrome (122, 167, 168). We hypothesize that in these forms of disorder the L3N alterations are not as robust. Here, the development of the L3N is probably affected later, after the age of 2.5 years, when further molecular maturation of the L3N occurs. Cognitive pathology is in such cases related to synaptic network architecture or/and fine molecular deflection at the level of individual synapse, without any dendritic and axonal changes that can be defined as pathological.

However, complex cognitive processing is disturbed in most cases of ASD at earlier stages of development (336, 337). Neurological and cognitive pathology is frequently present during the first year, sometimes even at birth (338, 339). In the most serious cases, there is ASD comorbidity with the intellectual developmental disorder (340) or/and epilepsy (341). In cases with absent comorbidity, many parents report a concern about socio-emotional interactions during the first year of life (342–345). Neuroimaging further revealed network inefficiencies during the first year of life (337, 346, 347). However, although specific ASD symptoms are not seen before the age of two, epidemiological and genetic studies support the idea, that alterations of neuronal development occur during the prenatal or perinatal time (348–355).

Thus, the “uniquely biphasic” pattern of dendritic growth of the L3N, with delayed structural and molecular maturation during the post-infant period (after 1.5 years), indicates that this neuronal class is particularly vulnerable during the perinatal period, when harmful events related to ASD are expected to occur. Pathological alterations induced at that stage may not be severe enough to affect functional demands of the L3N at the early postnatal period. However, the perinatal lesion could manifest after the second year, since the role of L3N, in now much complex microcircuitries, becomes more demanding at this time. In this view, the earlier “perinatal lesion” becomes visible as notable functional impairments after the second year (327, 356).

Most ASD cases will be diagnosed earliest by the age of 18 months (103). However, social cognition is often altered earlier, even in infants that do not show neither neurological, nor intellectual impairment. Retrospective studies have shown that ASD affected children clearly have different behavior related to social responses by 12 months, like gazing in a single direction and the way how they respond to their name (357). On functional magnetic resonance imaging (fMRI) altered connectivity in ASD was found by the 6 months (127, 346) and EEG signal was found to be altered by the 3 months (358). Above mentioned behavioral and functional aberrations present at early stages of infancy (337), further suggest that ASD symptoms are related with structural alterations from the early postnatal period. Interestingly, EEG pathology of ASD is first observed in the left temporal electrode (T7), and the frontal lobe starts to differ between 15 and 18 months of age, which corresponds to the beginning of the second L3N growth spurt. The developmental (“biphasic”) pattern of L3N, with intensive perinatal dendritic growth (suggesting high vulnerability), and second growth spurt around the age of 2, corresponds to the timing of alterations in cortical functioning and appearance of symptoms in infants with ASD. Understanding relations between L3N development and functional changes of cortical activity is important in early detection of ASD and might help to develop algorithms as combination of functional imaging methods and focused behavioral testing.

## Gene-Environment Interaction During Postnatal Development Affects Associative Circuitry Architecture And May Contribute to Autistic Traits

Architecture of the mature cortical network is determined through complex gene-environment interactions during intricate developmental steps (1, 260, 330, 359–365). Given the unimaginable number of possible interactions between genes and environment, there are large interindividual differences in the size of particular areas, and even in internal cytoarchiteconics, particularly within the prefrontal cortex (77, 366–368). Large interindividual differences were found among the dendritic structure of cortico-cortical projecting neurons in high associative areas of the human brain, correlating with the level of education (369). All mentioned interindividual differences point to a strong environmental impact on cortical development.

The mechanism of developmental plasticity leading to interindividual differences in cytoarchitectonics and neuron morphology is related to synaptic overproduction. By activity-dependent molecular tuning of synaptic spines, it is determined which synapse will remain and which will be removed from the network during the pruning process (370, 371). This model is defined as selective stabilization hypothesis (318) and proposes that through synaptic tuning the environment is shaping the architecture of the neural network. The highest degree and longest period of synaptic overproduction are so far described for the L3N in the prefrontal cortex, particularly at dendritic compartments (distal side branches of apical dendrite) targeted by cortico-cortical and intra-cortical projections (285). Altogether, psychological, educational, social and emotional milieu has a predominant influence in reshaping circuitries which are involved in processing the most complex cognitive functions (29, 76, 226, 309, 332, 372–379).

Taken together, these findings suggest that human-specific cognitive functions and circuitry specializations (1) have foundation in interactions between genes (25, 380–382) and environment (242, 363, 383, 384) during the period of synaptic overproduction and pruning. In humans, the period of synaptic overproduction and pruning is the period of the highest magnitude of acquisitions of new knowledge. It might look paradoxical that this occurs with a decrease in the number of synapses, but the final functional outcome of pruning leads to increased connectivity of the cortical network. Therefore, this process allows humans to acquire the highest level of cognition, but it also prolongs the vulnerability period, increasing the chance for the formation of abnormal circuitry (296, 306, 327, 364, 385–387).

Such developmental reshaping could be an important factor in the development of ASD, particularly in subjects with a mild form of the autism spectrum phenotype. Recognition that ToM deficit is a core psycho-pathological substrate of ASD, has allowed better diagnosing of both patients with mild impairment and individuals with atypical symptoms or personality traits associated with ASD, which fall under the broad autism phenotype (388–390). It is possible that the “pathological” substrate can be found only in the circuitry architecture, without any structural or molecular impairment of neurons and their pathways. In line with this possibility is a higher incidence of ASD in school rather than preschool children (391), suggesting that in some cases, “autistic” circuitry architecture is established through late childhood, or even adolescence (103, 392, 393). So, the broad or subthreshold autism spectrum conditions, could be used as a model to understand trajectories of “nature-nurture” interactions, guiding neurodevelopment toward, or away from ASD.

We propose that in such individuals, the emotional and psycho-social environment during infancy and childhood is crucial for the appearance of ASD or autistic traits (387, 394, 395). Vice versa, in individuals with genetic backgrounds to develop ASD or autistic traits, this opens a huge window of opportunity for cognitive rehabilitation, particularly considering the highly extended period of circuitry reorganization in the human prefrontal cortex. At an epidemiological level, mild and atypical cases should be far more numerous then serious cases of ASD, and are therefore of much higher societal impact. Second, enhanced emotional and psychological interaction in infants and children, have been shown to have a stimulating effect on the development of ToM (396, 397), suggesting that those with mild symptomatology and subtle alterations in circuitry organization have a greater chance for a positive outcome of early intervention (398).

## Overview of Reported Cortical Neuronal Pathology in Autism

Despite pathological changes observed in the cerebellum, amygdala and brainstem (399–402), imaging data and studies on post-mortem material are implying that cerebral cortex circuitries are the most plausible candidate to produce core deficits of autism. However, specific cortical neuronal alterations are yet to be described and present data are often contradictory. Some ASD cases have reduced neuronal cell body size but increased number of minicolumns and increased neuronal density (403, 404). These findings suggest an increased number of neurons in frontal, temporal and parietal regions of ASD cortices (405, 406). In other ASD cases, neuron numbers and density were unchanged (407), while some have a reduction in the number of neurons (408). Cellular patches were found in prefrontal and temporal cortices of ASD patients, while again no change in in neuron density was described (409).

Cell body size was unaltered in the dorsolateral medial prefrontal cortex (405), superior temporal gyrus (407), anterior cingulate cortex (410), hippocampus (411), and amygdala (412). Smaller cell size was found in cortical regions with identified minicolumnar pathologies in ASD, i.e., frontal regions (403, 406, 413), as well as primary motor, sensory and visual cortices (414). In the fusiform gyrus (415), hippocampus (416) and portions of the anterior cingulate cortex (417, 418), smaller neuron cell bodies of varying types have also been reported. These changes in cell body size are considered to be present in preadolescent stages between ASD and controls, and this effect becomes diminished later on (400, 410). Importantly, changes in cell body size are usually related with changes in dendritic morphology. However, Golgi studies on ASD neocortices, showed that there is no dendritic pathology in neocortical pyramidal and non-pyramidal neurons (411, 419), but increased density of dendritic spines was found in layer II of temporal, parietal and frontal region (420). Higher spine density suggests impaired synaptic pruning, and is correlated to decreased brain weight and lower levels of cognitive functioning in ASD (164, 421–424).

Reduction in neuronal size and loss of neurons in ASD suggests a bias in connectional abnormalities present in multiple areas of the association cortex, specifically within layers that are involved in long-range connectivity (406, 414). The alteration of neuronal classes essential to these circuitries is expected to be the main correlate of altered cognitive processing. In line with that, it was suggested that the total number of a special neuronal subtypes found only in species with highly developed social cognition, von Economo spindle cells, is decreased in autism, but stereological analysis in the frontal part of the insula could not confirm that assumption (425).

Based on the level of gene expression, a reduced number of distinct cell subtypes in layers IV and V, the calbindin and parvalbumin neurons, was suggested (426, 427). So far the only neuron-specific pathology documented histologically in ASD is a decreased number of parvalbumin interneurons in medial prefrontal cortex (428). Parvalbumin expressing cortical neurons provide inhibitory input to cortico-cortical projecting principal cells (429–431). The temporal pattern of change in axon terminals of parvalbumin interneurons parallels the changes in dendritic spine density on layer III principal cells (206, 432). The chandelier subpopulation of parvalbumin neurons, which is projecting to axon initial segment of principal neurons, is found to be affected in prefrontal cortex of ASD subjects (433). Therefore, decreased number of parvalbumin neurons in ASD may be related to alterations of postnatal refinements in cortical circuitry related with associative pyramidal neurons.

In conclusion, despite no direct evidence of L3N pathology in ASD being found, already mentioned findings that in ASD there is a higher spine density in layer II (420), could suggest an altered synaptic pruning of projections arising from associative L3N.

## Disclosure of Pre-Existing Lesion Through Late Maturation of Associative Neurons in Autism

In this manuscript we present an interesting observation about neuron pathology of an ASD case from Zagreb's Neuroembryological Collection (434–436), and evaluate the possibility that appearance of ASD symptoms is correlated with maturation of associative L3N during early childhood, but without disruption of their development.

**Figure 11:**
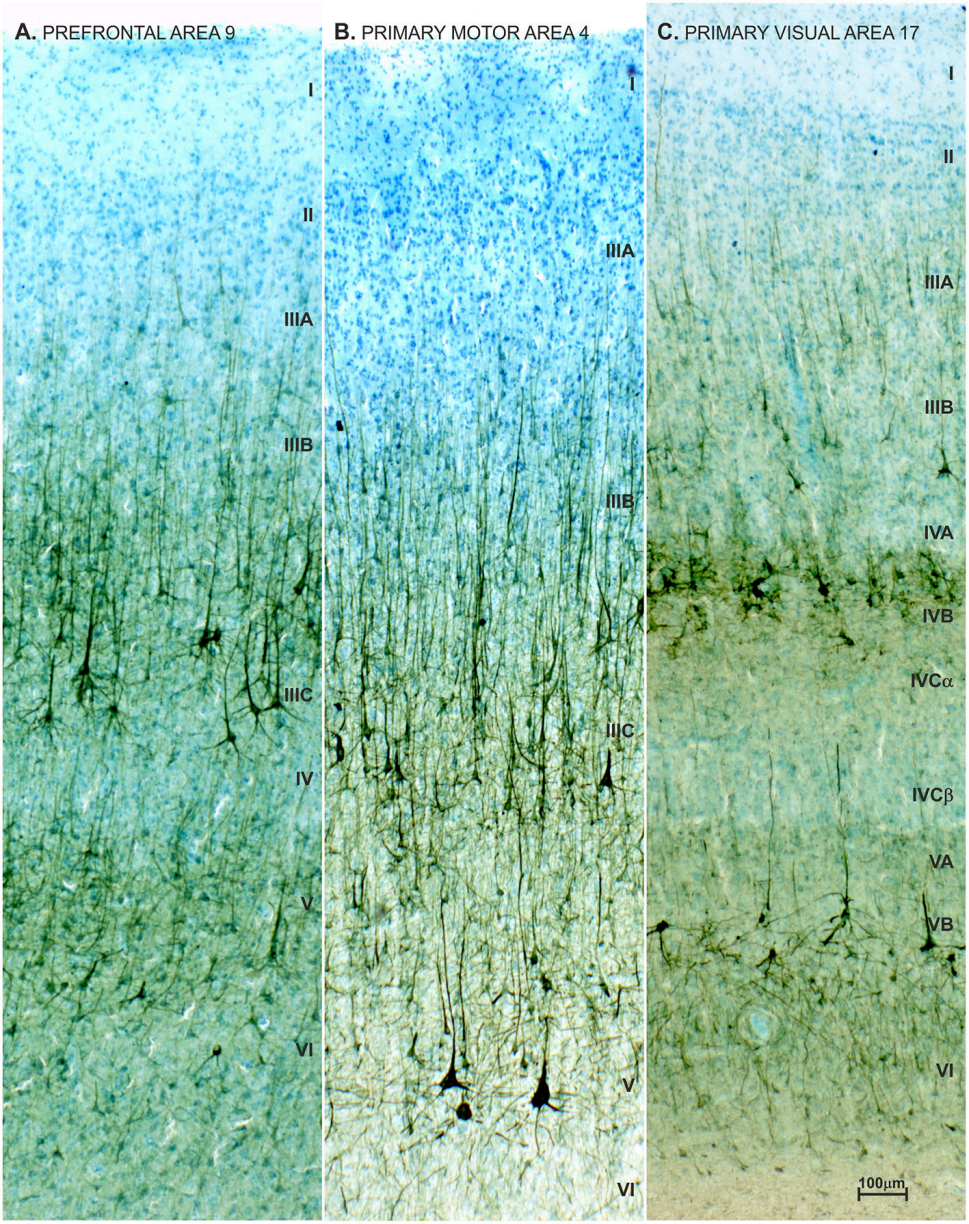
Neurofilament staining (SMI32) in neocortex of adult female with autism. Pattern of SMI32 immunostaining counterstained with Giemsa in the prefrontal area 9 **(A)**, primary motor area 4 **(B)** and primary visual area 17 **(C)**, in a 21-year-old autistic female with a comorbidity in the form of epilepsy and intellectual disability (according to DSMIII classification). The analyzed material is a part of Zagreb's Neuroembryiological Collection (434–436). The distribution, density and level of neurofilament (SMI32) expression did not differ from normative control (81, 438), and no obvious disruption of cytoarchitecture was observed (68, 266). SMI32 stained sections were compared to normative control specimens obtained from the Zagreb Neuroembryological Collection, which includes 29 specimens, with an age span of 19 to 57 years.

**Figure 12:**
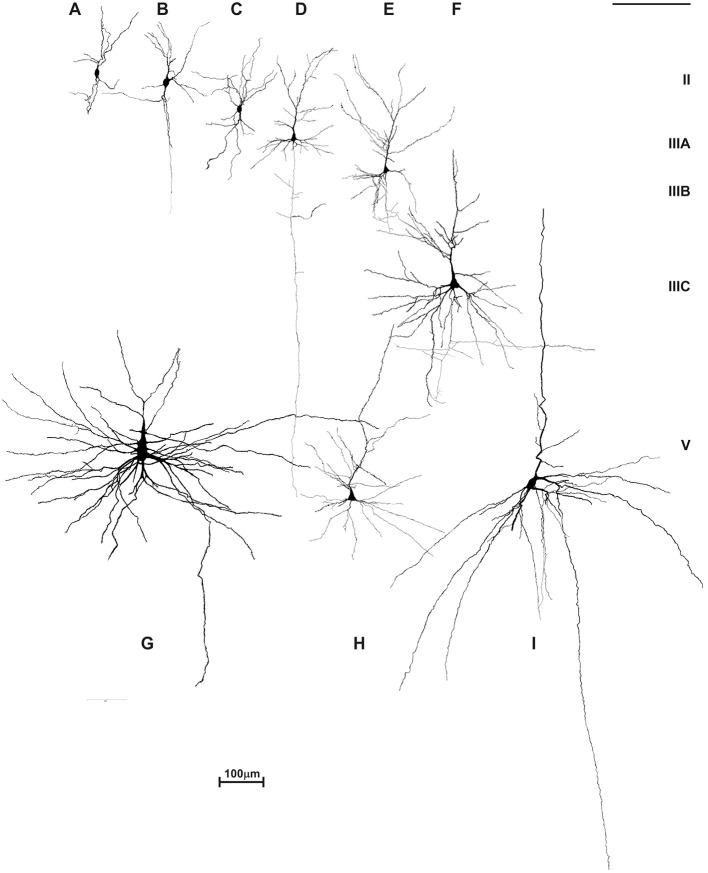
Reconstructions of rapid Golgi impregnated principal neurons in the neocortex of an adult female with autism. Neurons are marked in order of their laminar position: atypical pyramid-like cells **(A–C)** and typical pyramidal cells in analyzed neocortices were shown: layer II **(D)**, layer IIIA/B pyramidal cells **(E)**, layer IIIC pyramidal cells in prefrontal cortex **(F)**, giant Betz pyramidal cells in layer V of motor cortex **(G)**, small pyramidal cells of layer V **(H)** and large Meynert pyramidal cells of layer V/VI in area striata **(I)**. Atypical pyramid-like cells **(A–C)** were mainly located in the layer II, and some in the layer IIIA, but not in any other layers. They have oval cell bodies with bipolar orientation of dendrites, low spine density, and axons directed toward white matter. Such cell types were not observed in Golgi sections of a normal, adult human cerebral cortex. Golgi sections from this case were compared to 29 rapid Golgi stained specimens, with an age span of 19 to 57 years. No other qualitative signs of dendritic, nor spine pathology and density could be found on rapid Golgi slices, and morphology, cell body size, dendritic extent, and complexity **(D–I)** corresponded to pyramidal neurons of the same laminar position in an aged-matched controls.

We performed an in depth analysis of brain tissue from a 23-year-old female with ASD (based on DSM-III-R criteria) (437), with mild intellectual disability and epilepsy. We did not observe changes in the brain's gross morphology, cytoarchitectonic structure, nor expression of non-phosphorylated-neurofilament H (SMI32) which is highly expressed in L3N (Figure 11). Unchanged intensity of neurofilament staining (81, 438) suggests normal axonal development of associative neurons. On rapid Golgi impregnated sections of prefrontal cortex (Brodmann area 9), primary motor cortex (Brodmann area 4) and primary visual cortex (Brodmann area 17), we did not observe changes in dendritic size or in spine density of L3N or other classes of principal neurons. However, a small fraction of neurons in the layer II and upper part of layer III in the analyzed areas exhibited abnormalities of dendritic morphology (Figure 12).

**Figure 13:**
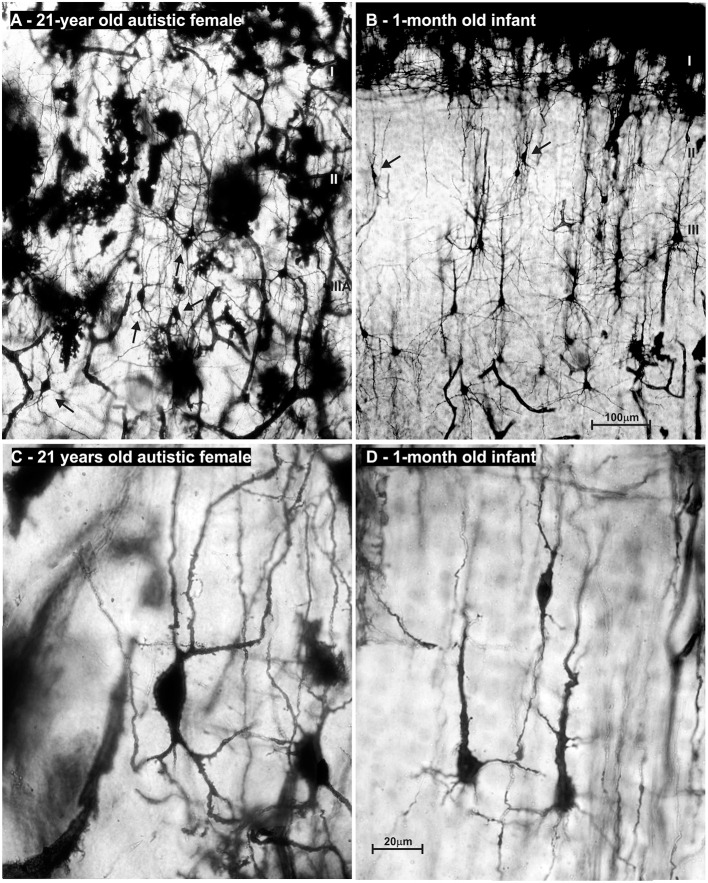
Microphotography of rapid Golgi sections in the prefrontal cortex of an adult female with autism, compared to a normal 1-month-old infant. On low power microphotography of layer II/IIIA in the prefrontal cortex of a 21-year-old autistic female, numerous bipolar—pyramid-like cells were found **(A)**. In the prefrontal cortex of a normal 1-month old infant **(B)**, numerous neurons with similar morphology were found in the same position (arrows). High power microphotography shows layer II bipolar-pyramid like cells in the analyzed subject **(C)**. Note that dendrites had spines, but their density was low. Upper dendrites were directed to the layer I, resembling apical dendrite morphology. On the other pole, two dendrites resembled basal dendrite morphology. An axon arose from one of those dendrites and was directed to the white matter. In the prefrontal cortex of a newborn infant, immature pyramidal neurons with similar morphology are found **(D)**. In the adult autistic subject neuron morphology of spiny bipolar neurons regarding cell body shape, and dendritic complexity is similar only larger when compared to healthy neonate. The presented material is a part of Zagreb's Neuroembryological Collection, which also includes normative control specimens of 31 rapid Golgi specimens, lifespan from infant to adolescent. Scale bar indicates 100 μm **(A,B)** and 20 μm **(C,D)**.

Alongside well developed and regularly oriented principal neurons, a subset of neurons in layers II/IIIA had spiny dendrites, whose morphology resembled those of immature principal cells (Figure 13A). Such dendritic morphology with low spine density is characteristic for developing principal neurons at initial stages of their dendritic differentiation. Developing neurons with similar morphology are found in the neocortex of healthy neonates (Figure 13B), but not later on. Since only part of the neurons had the immature dendritic morphology (Figures 13C,D) (204, 439, 440), we concluded that in the analyzed subject a selective fraction of cortico-cortically projecting neurons is affected. In particular, layer II and upper part of layer III contain cortico-cortical neurons that have relatively short axons, and participated in local microcircuits between neighboring areas (44, 74, 441–445).

Thus, their abnormal development in ASD may be a result of harmful events (including those induced genetically) occurring during the perinatal period. In this manner, development of microcircuitries established by short cortico-cortical neurons would be stalled at the neonatal stage. Importantly, this neuron class is not expected to go through distinct structural complexification after the first year. Therefore, we hypothesed that they will achieve adult structure by the age of 1, but full functional capacity will be achieved through maturation of associative neurons and related circuitries during early childhood (2–6 years). By having subtle alterations of the short cortico-cortical network, first symptoms, in general, would not appear before final maturation of local intracortical connections which occur later during childhood. It means that development of L3N and their projections in ASD could take a fully regular course, but may trigger appearance of symptoms.

Neurodevelopmental model with early structural lesions and a delayed appearance of symptoms is already established for schizophrenia (385, 446). Typical schizophrenia symptoms occur predominantly during late adolescence or early adulthood. Such timing is linked to massive synaptic pruning in the prefrontal cortex that occurs as part of normal development. So, in schizophrenia the appearance of symptoms is not a result of disrupted development at that time (90, 99, 447, 448). In fact, events occurring through the course of regular development are a trigger which may cause an already present, but for a long time asymptomatic impairment, to become eminent. Direct evidence for such a hypothesis comes from patients with metachromatic leukodystrophy, a disorder characterized by demyelination present at birth. The lesion remains without exacerbation up until late adolescence, when a schizophrenia-like psychosis will emerge (449, 450). So, a fixed “lesion” from earlier in development has been silent for decades, and interacts with normal brain maturational events that manifest much later in life. Despite the causative process not being obvious, it is still present long before the symptoms appear and any diagnosis is made (327, 360, 451–454).

## Development of Associative Neurons During Childhood and Relation to ASD Symptoms: Altered Development or Trigger For Pre-existing Lesion?

In this manuscript, we hypothesize that selective alteration of a specific subset of principal neurons could lead to global changes in cortical network connectivity. We applied this model to the ASD and social (pragmatic) communication disorders, which include disrupted social and communication functioning, with more or less severe global disconnectivity.

**Figure 14:**
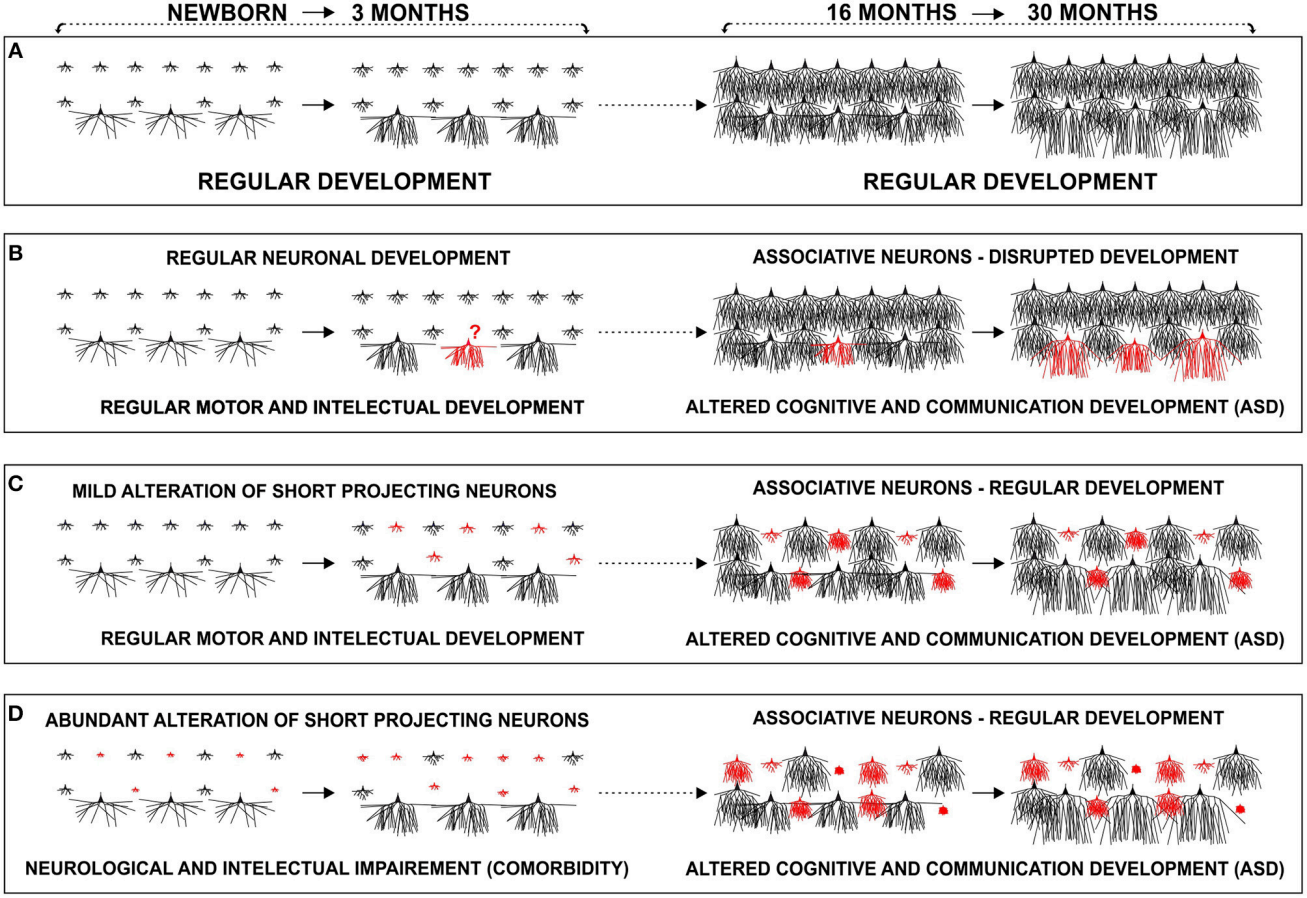
Proposed developmental models of cortico-cortical neuron alterations in autism: schematic representation of changes in dendritic morphology of layer III in the human prefrontal cortex during the early postnatal period (newborn–3 months) and around the second year (16–30 months). **(A)** In a typically developing infant, associative neurons undergo intensive dendritic differentiation during the first 3 postnatal months, whereas other classes of cortico-cortical projecting neurons, undergo major dendritic growth later during their first postnatal year. A late, second growth spurt, with large dendritic elongation in the period from 18–30 months, is so far described only in associative neurons. We propose that maturation of associative neurons during early childhood (1.5–6 years), plays a major role in global, functional changes of the cortical network, related with tremendous cognitive development. **(B)** Disrupted development of associative neurons (red), around age of 2, does not allow enhancement in inter-columnar and inter-areal connectivity, leading to cortical network impairment. Development of associative neurons does not have to be disrupted **(C,D)**. There might be a preexisting (perinatal) alteration, in a subset of short cortico-cortical neurons (red) that would become evident around the age of 2, during typical maturation of intra-cortical connections established by associative neurons. **(C)** In the case of mild perinatal disruption of short cortico-cortical neurons, the lesion would remain functionally silent until late differentiation of associative neurons, whereas in **(D)** cases where alterations are more robust, comorbidities in form of neurological, and intellectual impairment, manifest earlier. We suggest that alteration can be present in various forms thus creating many phenotypes of ASD.

We propose that contrary to normally developing children (Figure 14A), there might be a disrupted development of inter-collumnar connectivity within the prefrontal cortex of ASD patients, as these microciruitries undergo intensive maturation in the period between 2 and 6 years when ASD manifests. Associative L3N, which are the main source of local excitatory cortico-cortical connections, and are thus responsible for inter-columnar connectivity, undergo intensive structural and molecular changes during the same time (Figure 14B). Disrupted maturation of intracortical connectivity may then consequently alter outputs from the prefrontal cortex. The severity of this pathology would depend on the extent and timing of disruption within those microcircuitries.

Lacking evidence for structural pathology of the L3N in ASD both in our reported case and overall in the literature, opens the possibility that differentiation of this neuron class takes a regular course. Thus, their developmental incorporation into maturing circuits during childhood will reveal a pre-existing (perinatal) lesion in other neuronal classes and microciruitries. Despite regular development of intracortical connections during the second year, and throughout the rest of early childhood, the cortical network will not be able to reach a new/higher level of information processing, as there is a pre-existing alteration in other classes of projection neurons, e.g., a subset of cortico-cortical neurons with short projections. We propose that in the case of a more subtle disruption of neurons, which are a source of short cortico-cortical circuitries, the lesion remains fully silent until the age of 2 (Figure 14C), whereas in the case of more robust alteration, neurological, and intellectual comorbidity may appear earlier (Figure 14D).

These two proposed models are also not mutually exclusive. Direct alteration of L3N or “disclosure” of pre-existing lesions on other neuronal classes during differentiation of associative neurons around the age of 2, could be present in different phenotypes of ASD, or even act at the same time (455, 456).

The important concept in understanding the mechanism of ASD is gene-environment interaction in shaping the architecture of the developing neuronal network (457, 458). The environmental factor may induce or prevent appearance of the ASD pathological functioning, like infection, malnutrition, toxins, or vascular insult (227, 354, 356, 459–462).

While not specifically recognized yet, structural and molecular alteration of mirocircuitry is clearly related with ASD, but in the subthreshold autism phenotype there might be “autistic architecture” of the cortical network, without evident structural or molecular pathology. It is intriguing that in such conditions, psychosocial ambience is exclusively related with appearance of autistic traits, particularly taking into consideration that associative and intracortical circuitries have the highest rate, and most protracted period of synaptic overproduction. Finally, the protracted period of highly plastic circuits involved in ASD pathology opens a new potential in rehabilitation strategies, particularly if early clinical detection approaches are applied (348, 351, 358, 463–472).

## Ethics Statement

This study was carried out in accordance with Croatian legislation and the approval of the Ethical Committee School of Medicine, University of Zagreb (number: 380-59-10106-14-55/152; class 641-01/14-02/01; 1 July 2014).

## Author Contributions

ZP, DS, and DD contributed equally to the work. ZP designed concept of research, performed Golgi experiments, analyzed Golgi and SMI32 sections, wrote the manuscript, prepared figures. DS analyzed Golgi and SMI32 sections, performed quantitative analysis of neuron morphology in the neonate, prepared figures, wrote and structured the manuscript, and the literature. DD analyzed Golgi and SMI32 sections, wrote and revised the manuscript, and the literature. AH interpretated the data, revised the manuscript. MRR reconstructed Golgi impregnated neurons, revised the manuscript. NJ-M analyzed Golgi and SMI32 sections, performed immunohistochemical experiments, revised manuscript. All authors read and approved the final version.

### Conflict of Interest Statement

The authors declare that the research was conducted in the absence of any commercial or financial relationships that could be construed as a potential conflict of interest.
